# Sex-Specific Impact of Inflammation and Nutritional Indices on AVF Blood Flow and Maturation: A Retrospective Analysis

**DOI:** 10.3390/diagnostics15101278

**Published:** 2025-05-18

**Authors:** Özgür Akkaya, Ümit Arslan

**Affiliations:** 1 Department of Cardiovascular Surgery, Faculty of Medicine, Alanya Alaaddin Keykubat University, Antalya 07450, Türkiye; oakkaya369@gmail.com; 2 Department of Cardiovascular Surgery, Faculty of Medicine, Ataturk University, Erzurum 25030, Türkiye

**Keywords:** arteriovenous fistula, chronic kidney disease, sex differences, neutrophil-to-lymphocyte ratio, systemic inflammatory index, systemic inflammation response index, prognostic nutritional index, Doppler ultrasound

## Abstract

**Background**: Arteriovenous fistula (AVF) failure rates are consistently higher in females, although the underlying mechanisms remain incompletely understood. Inflammatory processes play a key role in AVF remodeling and venous arterialization, yet their influence may differ by sex. This study aimed to evaluate the impact of inflammatory indices on AVF blood flow and maturation, with a focus on sex-specific differences. **Methods**: This retrospective analytical study included 110 patients (50 females, 60 males) undergoing initial surgical AVF creation. Postoperative assessments occurred at the fourth and sixth weeks. Patients demonstrating insufficient maturation (blood flow < 600 mL/min) at the fourth week were re-evaluated after two weeks without any intervening procedures or additional interventions. **Results**: Intraoperative Transit-Time Flow Measurement (TTFM) revealed significantly higher median AVF blood flow in males compared to females (289 mL/min vs. 200 mL/min; *p* < 0.001). Doppler ultrasonography (DUS) findings confirmed these sex-related differences, demonstrating consistently lower blood flow rates in female patients. An elevated neutrophil-to-lymphocyte ratio (NLR) was associated with approximately a 31% reduction in AVF blood flow among females, whereas an increased C-reactive protein-to-albumin ratio (CrA) correlated with an approximate 9% decline. In males, an elevated systemic immune-inflammation index (SII) and systemic inflammation response index (SIRI) were significantly associated with decreased AVF blood flow. Conversely, a higher prognostic nutritional index (PNI) positively correlated with AVF blood flow in both sexes. Risk factors associated with inadequate AVF maturation (<600 mL/min at sixth week) included female sex, advanced age, obesity, smoking, anemia, low vitamin D levels, and elevated inflammatory indices (NLR, SII, and SIRI). **Conclusions**: Inflammatory and nutritional indices derived from routine laboratory tests may assist in estimating AVF maturation likelihood. While DUS reliably assesses AVF blood flow, complementary evaluation methods may be required to assess the broader vascular status. Further research is needed to clarify sex-specific inflammatory mechanisms influencing AVF outcomes and to guide individualized management strategies.

## 1. Introduction

An arteriovenous fistula (AVF) is a surgically created vascular access that establishes a direct arterial-to-venous connection, serving as the optimal conduit for hemodialysis (HD). Effective HD necessitates sufficient AVF maturation, characterized by reliable cannulation and sustained high blood flow rates [1]. According to the ‘rule of sixes’, a mature AVF requires a minimum blood flow rate of 600 mL/min, a diameter of at least 6 mm, and a depth not exceeding 6 mm below the skin surface [2]. Dember et al. [3] further defined maturation as the capacity to consistently deliver adequate dialysis clearance over at least three hours per session, sustained over four consecutive weeks, with a pump flow rate of 300 mL/min. In clinical practice, Doppler ultrasonography (DUS) is indispensable for assessing maturation and detecting AVF dysfunction [4]. While a palpable thrill or detectable venous pulse frequently indicates sufficient maturation, standardized routine DUS criteria remain lacking [5]. Despite the significant utility of DUS, a critical need for cost-effective, non-invasive, and rapid predictive markers of maturation remains, especially in resource-limited settings where ultrasound availability may be restricted. Numerous investigations notwithstanding, the universally accepted criteria to define AVF maturity remain elusive [6]. Factors including fistula age, vessel caliber, wall tension, uremia, hypoxia, and systemic diseases are well-established contributors to AVF dysfunction [7,8]. Nonetheless, inflammation following vascular interventions is inevitable, prompting growing interest in inflammatory and molecular biomarkers as predictive tools for AVF maturation and potential failure [9,10].

Fistula maturation involves a complex vascular remodeling process exhibiting considerable individual variability and notable sex-related differences [11,12]. In females, AVF maturation frequently presents delays, lower success rates, and an increased requirement for subsequent interventions [13]. This sex-specific disparity may arise from distinct inflammatory responses mediated by genetic and hormonal influences, significantly impacting venous remodeling and adaptive vascular changes [14,15,16]. Consequently, delayed AVF maturation contributes prominently to the higher prevalence of central venous catheter (CVC) usage among women at hemodialysis (HD) initiation [17]. The maturation and ultimate patency of an AVF are determined by intricate multimolecular inflammatory interactions [18]. Within this complex interplay, inflammatory biomarkers derived from routine laboratory tests, particularly dual or triple-cellular indices, have gained prominence for predicting AVF outcomes [19]. This study aimed to assess the predictive value of the systemic immune-inflammation index (SII), systemic inflammation response index (SIRI), prognostic nutritional index (PNI), neutrophil-to-lymphocyte ratio (NLR), and C-reactive protein (CRP)-Albumin ratio (CrA) on AVF blood flow (mL/min) measured by DUS and AVF maturation adequacy. Particular focus was directed toward assessing sex-related differences in these predictors among patients scheduled for HD within three months who underwent surgical AVF creation for the first time.

## 2. Materials and Methods

### 2.1. Study Design and Patient Selection

This retrospective analytical cohort study included 110 patients who were scheduled to initiate hemodialysis (HD) within three months and were referred from the nephrology department to our surgical unit for their first AVF creation. Demographic and clinical data were obtained from a systematically maintained hospital-based database comprising prospectively recorded physician notes and administrative information.

### 2.2. Inclusion and Exclusion Criteria

Patients aged ≥ 18 years who underwent first-time AVF creation, had no pre-existing catheters, had not yet initiated hemodialysis, presented to the cardiovascular surgery outpatient clinic at the fourth and sixth postoperative weeks, and had available Doppler ultrasound records were included in the study.

Patients with existing HD catheters, those requiring urgent HD initiation via catheter placement, or individuals with a prior history of AVF creation were excluded. Patients with a history of kidney transplantation were also not included. To minimize confounding effects associated with inflammation, individuals with autoimmune diseases (e.g., systemic lupus erythematosus, rheumatoid arthritis, vasculitis) or those receiving immunosuppressive therapy for any reason were excluded. Patients whose AVF procedures were canceled due to active infection or who demonstrated clinical signs of active infection during follow-up were also excluded. To ensure consistent AVF flow measurements, patients with an ejection fraction below 45%, a recent history (within the past three months) of myocardial infarction, a history of cerebrovascular disease (e.g., stroke or transient ischemic attack), active malignancy, sepsis, hematological disorders, or those who had undergone major surgery within the recent period were excluded.

### 2.3. Perioperative Assessments and Surgical Approach

Alongside standard preoperative laboratory evaluations, additional parameters including iron, vitamin D, CRP, ferritin, and uric acid levels were recorded. Since 2019, all Doppler ultrasonography (DUS) evaluations and surgical procedures in our clinic have been performed by cardiovascular surgeons with a minimum of 15 years of experience and formal training in vascular ultrasonography. This consistent operator expertise significantly minimized both intra- and inter-operator variability. To identify the optimal site for AVF creation, DUS was performed using a Mindray system (Mindray Biomedical Electronics Co. Ltd., Shenzhen, China). In general, the non-dominant upper extremity was preferred. For radiocephalic (RC) AVFs, a minimum inner diameter of 2.0 mm for both the artery and the vein was required. For brachiocephalic (BC) AVFs, a vein diameter of ≥3.5 mm was considered suitable [20,21]. The anesthesia technique—either nerve block or local anesthesia with sedation—was selected based on each patient’s clinical condition. Following end-to-side surgical anastomosis, intraoperative blood flow measurements were obtained from the venous segment using Transit-Time Flow Measurement (TTFM; MediStim VQ 1101, MediStim ASA, Oslo, Norway). The TTFM device provided detailed flow curves and quantitative parameters, including mean blood flow (mL/min), pulsatility index (PI), and diastolic filling percentage (%DF). Anastomotic insufficiency was defined as %DF < 50% and/or PI > 5, which prompted a critical intraoperative evaluation to determine whether immediate surgical revision was necessary.

### 2.4. Postoperative Follow-Up

Patients underwent postoperative evaluations at the cardiovascular surgery outpatient clinic at the fourth and sixth weeks. In addition to routine laboratory tests, AVF blood flow was measured and recorded by cardiovascular surgeons via DUS. Volumetric blood flow (Q) was initially calculated in cm^3^/s and subsequently converted into mL/min by multiplying the obtained value by 60 (since 1 cm^3^ equals 1 mL and 1 s corresponds to 1/60 min), as follows:

Q = V × A

(V) is the blood flow velocity (cm/s) measured by DUS, and (A) represents the cross-sectional area of the vessel [22]

A = π × (D ÷ 2)^2^

(D) is the vessel diameter.

At the fourth-week assessment, patients exhibiting AVF blood flow ≤ 500 mL/min received supportive interventions such as exercise programs, nutritional optimization, anemia management, glucose and lipid profile adjustments, and iron supplementation [23,24,25]. By the sixth postoperative week, secondary interventions—including interventional or surgical revision—were indicated for patients with critically low blood flow (≤200–250 mL/min) and those with flow rates between 400 and 500 mL/min who failed to achieve adequate suction during HD. Advanced therapeutic strategies exceeded the scope of this study and thus were not evaluated. Inflammatory and nutritional indices (SII, SIRI, PNI, NLR, and CrA) were calculated from routine blood parameters according to previously established formulas detailed in Table 1 [9,26,27].

### 2.5. Outcomes

As all patients were in the predialysis stage at the time of evaluation, functional maturation, such as successful cannulation or dialysis adequacy, could not be assessed. Therefore, arteriovenous fistula (AVF) maturation was defined based on a Doppler-derived blood flow ≥ 600 mL/min, in reference to the 2006 Kidney Disease Outcomes Quality Initiative (KDOQI) guidelines, which proposed this threshold as an indicator of sufficient hemodynamic development prior to dialysis initiation [2]. Primary outcomes were analyzed in association with baseline inflammatory indices stratified according to sex, intraoperative TTFM data, and postoperative laboratory parameters.

## 3. Statistical Analysis

All statistical analyses were performed using SPSS (version 28, IBM, Chicago, IL, USA), Jamovi (version 2.6.19; The Jamovi Project, Sydney, Australia), and G*Power (version 3.1.9.7, Heinrich Heine University, Düsseldorf, Germany). Categorical variables were presented as frequencies and percentages, while continuous variables were expressed as mean ± standard deviation (SD) or median with interquartile range (IQR), depending on data normality. The Kolmogorov–Smirnov and Shapiro–Wilk tests were used to assess normality, and Levene’s test was employed to evaluate variance homogeneity. Comparisons between categorical variables were performed using chi-square or Fisher’s exact tests.

Continuous variables were compared using independent samples *t*-tests for normally distributed data and the Mann–Whitney U test for non-normally distributed data. When comparing continuous variables with heterogeneous variances, the Brunner–Munzel test was applied to provide a more accurate estimation of differences, especially when distributional assumptions were violated. Multiple group comparisons were conducted using one-way ANOVA or the Kruskal–Wallis test, according to data distribution. Repeated measures were analyzed using repeated measures ANOVA or the Friedman test, with Bonferroni correction applied to post-hoc comparisons.

To identify predictors of AVF blood flow, a generalized linear model (GLM) with a Gamma distribution and log-link function was utilized. This approach was selected due to its suitability in modeling positively skewed non-negative data, which is a common characteristic of blood flow measurements. Additionally, all continuous variables were standardized using Z-scores to improve model performance and ensure comparability across different scales. Standardization was particularly important for enhancing model convergence and reducing potential biases arising from differing measurement units. To ensure robust parameter estimation, 5000 bootstrap replications were performed, and associations were reported as Exp(B) values with 95% confidence intervals (CI). A *p*-value < 0.05 was considered statistically significant.

## 4. Results

The study cohort included 110 patients undergoing first-time AVF creation, comprising 50 females (45.5%) and 60 males (54.5%). The mean age was 50.1 ± 12.8 years (range: 25–70) for females and 54.8 ± 12.9 years (range: 23–75) for males (*p* = 0.061). Females exhibited a significantly higher body mass index (BMI) compared to males (mean difference: 2.03 kg/m^2^; *p* = 0.007). In contrast, no significant sex-related differences were observed regarding the prevalence of diabetes mellitus (*p* = 0.275) or hypertension (*p* = 0.540). However, coronary artery disease (CAD) prevalence was significantly higher among males than females (20% vs. 5.5%; *p* < 0.01). Concerning smoking status, 14 females and 23 males were active smokers at the time of evaluation.

**Figure 1:**
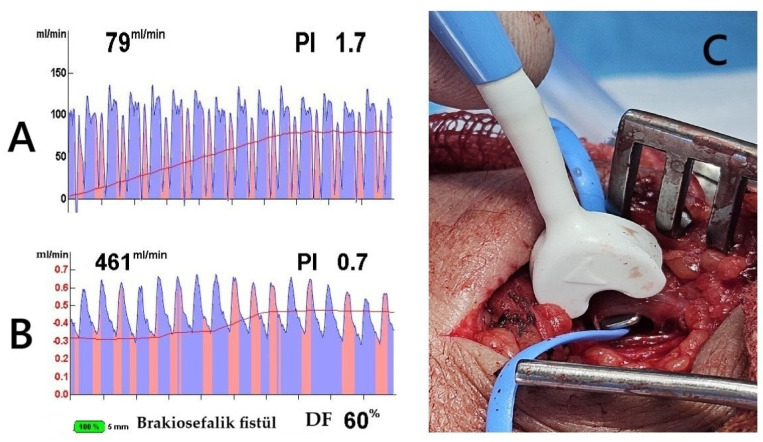
Transit-Time Flow Measurement (TTFM) and probe application. The images demonstrate TTFM use during brachiocephalic AVF creation in a 45-year-old male patient. (**A**) Measurement of the brachial artery before AVF creation shows adequate arterial flow. (**B**) After AVF creation, the probe placed on the vein (**C**) shows good flow measurements. The Pulsatility Index (PI) of 0.7 and Diastolic Filling (DF) of 60% indicate low resistance and effective blood flow continuity, essential for proper AVF maturation (brakiosefalik fistül; brachiocephalic fistula in English).

For radiocephalic fistulas, the mean arterial and venous diameters were 2.9 ± 0.6 mm and 3.1 ± 0.5 mm, respectively, in females, and 3.1 ± 0.4 mm and 3.2 ± 0.8 mm, respectively, in males. In brachiocephalic fistulas, females exhibited mean arterial and venous diameters of 3.9 ± 1.1 mm and 3.5 ± 0.8 mm, respectively, whereas males had corresponding measurements of 4.8 ± 0.5 mm for the artery and 4.0 ± 0.5 mm for the vein. The mean vascular diameters, calculated as the combined averages of both arterial and venous structures in the brachial and radial regions, were assessed. This approach was necessary because, among females, AVFs were predominantly created using the brachial artery (40 patients), with only 10 cases utilizing the radial artery. In contrast, the brachial artery was selected in 18 male patients due to inadequate radial circulation or small vein diameters (<2 mm). The arterial diameter was significantly greater in females than in males (3.8 ± 0.4 mm vs. 3.61 ± 0.9 mm; *p* = 0.013), whereas venous diameters were similar between sexes (3.3 ± 0.5 mm in females vs. 3.2 ± 0.4 mm in males; *p* = 0.118). Despite having larger arterial diameters, females exhibited significantly lower intraoperative blood flow rates compared to males based on TTFM measurements (median: 200 mL/min vs. 289 mL/min; *p* < 0.001). Additionally, females displayed higher vascular resistance, indicated by a greater PI (2.6 vs. 1.6; *p* < 0.001). These results highlight that AVF performance relies not only on vessel size but also heavily depends on individual vascular quality and blood flow dynamics (Figure 1A–C).

Preoperative laboratory assessments revealed significantly lower vitamin D levels in females compared to males (*p* < 0.001), along with more pronounced iron deficiency (mean difference: 14.8 µg/dL; *p* = 0.003). Females also exhibited significantly lower hemoglobin levels (mean difference: 0.8 g/dL; *p* = 0.002, Cohen’s d = −0.605), indicating a moderate clinical effect. Conversely, males demonstrated higher inflammatory indices, including elevated NLR (*p* = 0.040), SII (*p* = 0.172), and SIRI (*p* = 0.037), attributed to their increased neutrophil, monocyte, and platelet counts. In contrast, females displayed higher CrA values (*p* = 0.166), primarily due to their lower albumin levels. The preoperative and intraoperative characteristics of the patients are summarized in Table 2.

Inflammatory and nutritional indices were evaluated over time within each sex group. Table 3 presents the intra-group changes for males and females, respectively, highlighting the temporal trends in these parameters. According to the table, changes over time in biomarkers such as albumin, CRP, ferritin, and NLR were more pronounced and statistically significant in female patients compared to males.

During the postoperative period, persistently elevated neutrophil and CRP levels, together with lower albumin concentrations observed among females, led to distinct sex-related differences in inflammatory indices. The inflammatory parameters stratified by sex at the fourth and sixth postoperative weeks are summarized in Table 4. Although PNI values significantly increased within each group between the two follow-up evaluations, changes in other indices did not reach statistical significance. Notably, the decrease in CRP and the rise in albumin, which is particularly evident among female patients, may have favorably influenced the CrA index.

### 4.1. Predictors of Postoperative AVF Blood Flow (Functional Effect)

**Figure 2:**
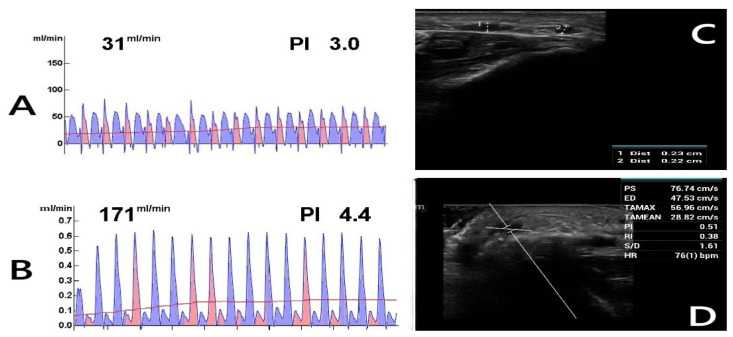
Postoperative Doppler ultrasound (DUS) assessment. The figure shows the postoperative evaluations of a 62-year-old diabetic female patient who continues to smoke. (**A**) TTFM measurement of the radial artery before anastomosis. (**B**) TTFM measurement of the radiocephalic fistula after anastomosis. (**C**) Fourth-week DUS examination reveals inadequate AVF maturation with a vein diameter of 0.23 cm (1), suggesting insufficient development. (2: Radial artery). (**D**) DUS parameters were measured as follows: PS (Peak Systolic Velocity): 76.74 cm/s, ED (End-Diastolic Velocity): 47.53 cm/s, TAMAX (Time-Averaged Maximum Velocity): 56.96 cm/s, TAMEAN (Time-Averaged Mean Velocity): 28.82 cm/s, PI (Pulsatility Index): 0.51, RI (Resistive Index): 0.38, S/D (Systolic/Diastolic Ratio): 1.61, HR (Heart Rate): 76 bpm. Blood flow (Q) was calculated using Q = TAMEAN × A × 60 [22], where A represents the vessel cross-sectional area. Given a fistula vein diameter of 0.23 cm, the calculated blood flow was 71.84 mL/min, indicating inadequate flow.

Postoperative DUS measurements revealed significantly higher AVF blood flow rates in males compared to females, as determined by the Brunner–Munzel test. At the fourth week, females had a 76.8% probability (95% CI: 0.68–0.85; *p* < 0.001) of having lower blood flow than males. While blood flow increased in both sexes by the sixth week, the improvement was more pronounced in males (mean difference: 255 mL/min for males vs. 199 mL/min for females). Consequently, the probability of females with lower blood flow further increased to 80.4% (95% CI: 0.71–0.89; *p* < 0.001) (Figure 2A–D).

Generalized linear regression models utilizing a Gamma distribution with a log-link function and Z-score standardization were performed to identify predictors influencing AVF blood flow changes. Negative predictors of blood flow included female sex, advanced age, high BMI, and smaller arterial diameter. Conversely, each unit increase in venous diameter enhanced blood flow by approximately 2.2%, while every incremental rise in intraoperative TTFM measurement improved blood flow by 28%. Clinical predictors of AVF blood flow at the fourth and sixth postoperative weeks are summarized in Table 5.

Except for PNI, all systemic inflammatory indices demonstrated inverse correlations with blood flow. Detailed analyses of the sex-stratified impact of inflammatory and nutritional indices on AVF blood flow were presented in Table 6. In females, elevated NLR significantly reduced blood flow by 31.3% at the fourth week (OR: 0.68, 95% CI: 0.40–0.98; *p* = 0.044) and by 31.4% at the sixth week (OR: 0.68, 95% CI: 0.345–0.954; *p* = 0.020). Although CrA exhibited a weaker negative impact compared to other indices, it was still associated with an approximate 9% decrease in blood flow at both the fourth (OR: 0.90, 95% CI: 0.78–1.28; *p* = 0.274) and sixth weeks (OR: 0.91, 95% CI: 0.72–1.34; *p* = 0.161), though these differences did not reach statistical significance.

Males were more adversely affected by higher SII and SIRI values compared to females. In males, elevated SII was associated with a 26.7% decrease in the probability of adequate blood flow at the fourth week (OR: 0.73, 95% CI: 0.4–1.13; *p* = 0.077) and a 15% reduction at the sixth week (OR: 0.85, 95% CI: 0.5–1.30; *p* = 0.304). Similarly, elevated SIRI significantly reduced the likelihood of achieving adequate blood flow by approximately 20.8% at the fourth week (OR: 0.79, 95% CI: 0.59–0.97; *p* = 0.049) and by 27% at the sixth week (OR: 0.73, 95% CI: 0.5–0.98; *p* = 0.005).

In contrast, higher PNI significantly increased the likelihood of sufficient AVF blood flow at the fourth week, with each unit increase raising the probability by 31.9% (OR: 1.31, 95% CI: 1.23–1.4; *p* < 0.001). However, by the sixth week, the positive effect of PNI diminished to approximately 13% per unit increase (OR: 1.13, 95% CI: 1.0–1.21; *p* < 0.001). Notably, no significant interaction between sex and PNI was observed at either the fourth (OR: 0.92, 95% CI: 0.80–1.05; *p* = 0.122) or sixth week (OR: 1.06, 95% CI: 0.96–1.23; *p* = 0.324), suggesting a consistent beneficial effect of nutritional status on blood flow across sexes. The observed reduction in the predictive value of PNI over time may reflect the natural postoperative recovery process, during which nutritional and inflammatory parameters gradually stabilize, thus decreasing the index’s sensitivity by the sixth week.

### 4.2. Predictors of Inadequate AVF (Structural Effect)

During the sixth-week DUS evaluation, AVF blood flow rates below 600 mL/min were categorized as inadequate maturation, while values exceeding this threshold indicated sufficient structural remodeling and adequate AVF function. Insufficient blood flow was detected in 20 females and 12 males, revealing a significant difference between sexes (*p* = 0.021). Among these 32 patients with inadequate maturation, 21 were active smokers; all had a BMI above 26 kg/m^2^ and arterial diameters below the optimal cutoff values—2.7 mm for women (Youden index = 0.814) and 2.1 mm for men (Youden index = 0.878).

ROC analysis was performed to determine the optimal intraoperative TTFM thresholds that best predicted adequate AVF maturation. The cut-off points were selected based on the maximum Youden index, identifying 153 mL/min for females (AUC = 0.936, 95% CI: 0.82–0.93; *p* < 0.001) and 169 mL/min for males (AUC = 0.947, 95% CI: 0.89–0.94; *p* < 0.001) as the most reliable discriminatory values. Interestingly, some female patients who achieved the intraoperative TTFM threshold yet presented inadequate postoperative flow exhibited deficiencies in vitamin D, iron, and hemoglobin, as well as elevated uric acid, LDL cholesterol, and triglyceride levels. Additionally, these patients displayed low adherence to medical treatments.

The primary endpoint in this study was the adequacy of AVF blood flow at six weeks. Post hoc power analysis indicated that the achieved statistical power was 0.70 (70%) at α = 0.05, falling short of the conventional 0.80 (80%) threshold for optimal power. Attaining 80% statistical power would necessitate a substantially larger sample, underscoring the importance of conducting future studies with larger patient cohorts to thoroughly evaluate predictors of AVF adequacy.

Binary logistic regression analysis revealed that several clinical factors were independently associated with an increased risk of inadequate AVF maturation. The multivariate binary logistic regression model demonstrated a good overall fit, as indicated by a McFadden’s R^2^ value of 0.596 and a Nagelkerke R^2^ value of 0.850, suggesting that the model explains a substantial proportion of the variance in AVF maturation outcomes. Female sex, advanced age, obesity, diabetes mellitus, hypertension, and active smoking emerged as significant negative predictors, with each contributing to a heightened likelihood of maturation failure. Additionally, lower hemoglobin levels (≤11.5 g/dL) and reduced vitamin D concentrations (≤22.7 ng/mL) were associated with a greater probability of unsuccessful AVF maturation. Inflammatory indices assessed at the sixth postoperative week demonstrated a strong association with AVF outcomes. Specifically, higher NLR, SII, and SIRI values independently predicted an increased likelihood of maturation failure, emphasizing the adverse role of systemic inflammation in early AVF remodeling. The negative predictors of AVF maturation are presented in Table 7.

Although CRP was negatively associated with AVF maturation initially (*p* = 0.024), its predictive power diminished by the sixth week (*p* = 0.189). Similarly, CrA significantly predicted inadequate AVF at the fourth week (estimate = −0.8; *p* = 0.01), but this relationship was no longer significant at the sixth week (*p* = 0.257), likely reflecting improvements in nutritional status and associated albumin elevation. Indeed, rising albumin levels over time strengthened the positive influence of PNI, enhancing its predictive power from a 1.2-fold increase at the fourth week to a 2.1-fold improvement at the sixth week (OR = 2.1, 95% CI: 1.6–3.0; *p* = 0.003).

Regarding vascular parameters, venous diameter was identified as being 3.7 (95% CI: 1.1–8.7; *p* = 0.035) times more influential in achieving adequate AVF maturation compared to arterial diameter. However, inclusion of intraoperative TTFM data into the predictive model diminished the significance of vessel diameters, reinforcing the superior reliability of TTFM as an independent predictor of AVF adequacy (OR = 1.28, 95% CI: 1.1–1.63; *p* < 0.001).

## 5. Discussion

This retrospective study demonstrated that blood flow measurements assessed by Doppler ultrasound (DUS) during the first six weeks after AVF creation significantly differed between sexes, and that these differences were further influenced by systemic comorbidities, inflammatory status, and nutritional conditions. At the end of the sixth postoperative week, 20 female and 12 male patients failed to achieve the 600 mL/min threshold recommended by KDOQI guidelines [2] (*p* = 0.021). Although both sexes exhibited improvements in AVF blood flow over time, male patients demonstrated an average increase of 50–60 mL/min greater than females, whereas female patients showed a lower degree of flow enhancement. In the analysis of factors affecting blood flow, higher NLR and CrA levels in female patients were associated with a reduction in AVF blood flow by approximately 30% and 9%, respectively. In contrast, elevated SII and SIRI levels in male patients led to flow reductions of at least 15% and 20%, respectively (Table 6). Moreover, these inflammatory combinations served as negative predictors for achieving the threshold blood flow required for successful AVF maturation, as demonstrated in Table 7.

An arteriovenous fistula (AVF) is a surgically created vascular connection in patients with renal failure, facilitating effective hemodialysis (HD) through repeated cannulation and sustained high blood flow. Its successful maturation, characterized by the adaptive arterialization of the vein, is primarily driven by hemodynamic forces such as wall shear stress and vortex flow, which stimulate structural remodeling across all vascular layers—particularly the endothelium—but these adaptive responses appear to be less favorable in females compared to males [28,29,30,31,32]. Lee et al. [33] reported that women have a significantly lower AVF success rate and a 46% higher failure risk compared to men. Miller et al. [15] suggested that early technical failure and thrombosis, rather than vessel diameter alone, may underlie these differences, possibly reflecting sex-related variations in vascular reactivity. As a minimum, we advocate for the use of intraoperative TTFM to support surgical success. de Winter et al. [34] demonstrated that RCAVFs with intraoperative flows ≤ 160 mL/min had a 3.03-fold increased risk of maturation failure. According to our sex-stratified ROC analysis, the optimal TTFM cut-off values for predicting AVF maturation were 153 mL/min for females and 169 mL/min for males. Variations across studies may reflect differences in vessel diameters, probe characteristics, patient demographics, or surgical techniques. Our findings highlight the potential value of integrating sex-specific intraoperative flow thresholds into AVF assessment algorithms.

Although sex disparities in AVF maturation are well established, the underlying mechanisms remain incompletely understood, with substantial variability across studies [17]. Even after adjusting for differences in dialysis care, female HD patients—particularly younger women—exhibit higher mortality rates compared to males [35,36]. Sex-specific variations in vascular pathophysiology, immune responses, and comorbidity profiles may contribute to adverse outcomes and impair AVF remodeling through inflammatory pathways [14].

The evolving nature of the human immune system, shaped by genetic, hormonal, and nutritional factors, complicates the identification of mechanisms driving AVF failure [37,38]. Female sex, recognized as an independent risk factor for AVF failure, is further compounded by smaller vessel diameters, altered platelet function, and increased neointimal hyperplasia [15]. Experimental evidence suggests that reduced estrogen levels attenuate vascular protective mechanisms, while testosterone deficiency promotes venous wall thickening and shear stress, highlighting the distinct vascular effects of sex hormones [36]. Estrogen maintains vascular homeostasis by enhancing nitric oxide production, promoting endothelialization, and inhibiting inflammatory adhesion molecules [39,40,41], and its deficiency in postmenopausal women may exacerbate inflammatory responses, contributing to higher AVF failure rates. Kudze et al. [38] further supported the role of sex-specific molecular pathways in AVF maturation by demonstrating elevated levels of proteomic markers related to venous remodeling, thrombogenicity, and inflammation in female mice.

Beyond intrinsic molecular and hormonal factors, pharmacological interventions may critically influence AVF remodeling. Agents such as statins, ACE inhibitors, vitamin D supplementation, and antiplatelet therapies modulate key processes like endothelial function, inflammation, and vascular tone. Among these, vitamin D plays a pivotal role by regulating vascular homeostasis and suppressing proinflammatory cytokines; impaired vitamin D metabolism in CKD patients contributes to vascular dysfunction [42]. In our study, a serum vitamin D level of ≤22.7 ng/mL was associated with a 30% increased risk of AVF maturation failure (OR = 1.3, 95% CI: 1.05–1.91; *p* < 0.01), and vitamin D levels were significantly lower among females (*p* < 0.001), potentially explaining higher AVF failure rates in women. Statins contribute to vascular remodeling by modulating inflammation, inhibiting VSMC proliferation, and suppressing immune cell chemotaxis [43]. The SHARP (Study of Heart and Renal Protection) trial [44] demonstrated that simvastatin plus ezetimibe reduced LDL cholesterol and major atherosclerotic events in CKD patients while supporting vascular access patency, with statin therapy particularly recommended for postmenopausal women [45]. Antihypertensive agents, including ACE inhibitors, ARBs, and CCBs, may further promote AVF maturation by enhancing endothelial function through modulation of the renin–angiotensin–aldosterone system [46]. Although the role of antiplatelet therapy in AVF patency remains debated, agents such as clopidogrel, ticlopidine, and aspirin have shown benefits in preventing thrombosis and neointimal hyperplasia [47,48]. However, due to the retrospective nature of our study and the inability to confirm consistent medication adherence, variables related to statin, antihypertensive, and antiplatelet therapies were not included in our regression model.

Although HD significantly improves survival, nearly 40% of patients with kidney disease do not survive beyond five years, with early AVF failure almost tripling the risk of death [49,50]. Given the critical role of AVFs as primary vascular access, regular monitoring through combined physical examination and DUS assessment is universally recommended [51]. Integrating palpation and auscultation findings with DUS enhances the reliability of AVF maturation evaluation at the creation site. However, current risk-scoring systems—primarily based on fistula location, vessel diameter, patient demographics, vascular mapping, and systemic disease profiles—are largely derived from single-center studies [8,52]. Recent insights into inflammatory biomarkers have highlighted the need to refine existing models, as physical examination and DUS, while effective locally, provide limited information about the broader vascular condition [53].

Clinicians have increasingly explored the predictive value of routine laboratory parameters for AVF outcomes. Biomarkers such as albumin, fibrinogen, CRP, D-dimer, hemoglobin, calcium, phosphorus, parathyroid hormone, and ferritin have been linked to AVF maturation, although substantial heterogeneity among studies limits their clinical utility [54,55,56]. Emerging markers—including matrix metalloproteinases, plasma gelsolin, hypoxia-inducible factor-2 alpha, interleukins, and antiphospholipid antibodies—show potential but remain restricted by high costs and complex measurement techniques [57,58,59,60]. Given the established role of inflammation in vascular remodeling, Kaller et al. [9] identified elevated inflammatory states and impaired nutritional status, assessed through composite inflammatory indices, as significant predictors of AVF failure, a finding later validated by immunohistochemical analyses [19].

Emerging evidence highlights a paradigm shift from isolated biomarker evaluation to composite indices derived from routine laboratory parameters, providing a more integrated reflection of inflammation and intimal hyperplasia relevant to AVF maturation [18,61,62,63,64]. Among these, three-cell indices such as the systemic immune-inflammation index (SII) and Systemic İnflammatory Response İndex (SIRI) have gained prominence for their ability to capture immune competence and systemic inflammatory burden [65,66].

Huang et al. [67] reported independent associations between elevated SII and SIRI levels and increased CKD incidence and all-cause mortality, findings corroborated by Lai et al. [68] in a large CKD cohort linking SII to cardiovascular and cancer-related mortality. Elevated SII levels have also been implicated in the progression of albuminuria, a marker of CKD deterioration and cardiovascular risk in diabetic populations [69,70].

Ren et al. [26] identified elevated SII and SIRI as significant predictors of HD access failure, with risk increases of 2.7- and 2.3-fold, respectively. Consistent with these findings, our cohort demonstrated that higher SII and SIRI levels were associated with a 1.5- and 2.3-fold reduction in AVF maturation rates, despite differences in sample size and follow-up period. Notably, these negative effects were more pronounced in male patients, potentially linked to higher platelet, neutrophil, and monocyte counts, as well as increased antiplatelet therapy use, coronary artery disease prevalence, and smoking-related inflammation [71]. Regardless of sex, tight control of cardiovascular risk factors—smoking, hypertension, diabetes, and obesity—remains critical for optimizing AVF outcomes (Table 7).

In addition, the neutrophil-to-lymphocyte ratio (NLR) has been associated with AVF outcomes. Pasqui et al. [62] and Wongmahisorn [72] identified NLR thresholds predictive of early AVF failure. In our cohort, sex-specific thresholds of 2.6 for females and 4.3 for males were established, with women above 2.6 exhibiting greater reductions in AVF blood flow. Elevated NLR was overall associated with a 1.38-fold decrease in maturation (*p* = 0.004), likely reflecting impaired neutrophil–endothelial interactions and maladaptive vascular remodeling during surgical manipulation

Malnutrition due to inadequate dietary intake remains a significant challenge in patients with kidney disease. Kang et al. [73] demonstrated that improvements in the Malnutrition–Inflammation Score (MIS) through nutritional support were associated with significantly better survival outcomes. Similarly, Noce et al. [74] reported improved inflammatory profiles in CKD patients receiving oral nutritional supplementation, emphasizing the beneficial impact of nutritional interventions on systemic inflammation.

The prognostic nutritional index (PNI), calculated from serum albumin and lymphocyte counts, serves as an integrated marker reflecting both nutritional and inflammatory status. Importantly, PNI has been shown to predict mortality more reliably than albumin or lymphocyte count alone across different age groups [75,76]. Its clinical utility stems from capturing the interplay between malnutrition and inflammation, offering a comprehensive assessment of patient status [77]. Kaller et al. [9] highlighted the protective role of higher PNI values against thrombosis and AVF maturation failure, with levels below 39 indicative of elevated risk. In our study, preoperative PNI levels did not differ significantly between sexes (*p* = 0.110). However, by the fourth postoperative week, PNI levels were significantly lower in females compared to males (mean difference = 3.0; *p* = 0.026). Nutritional follow-up was conducted through multidisciplinary monitoring programs involving nephrologists, renal dietitians, and outpatient clinic nurses, with adherence assessed through both patient self-reporting and objective laboratory markers, including serum albumin, iron, hemoglobin, and PNI [78]. Following nutritional interventions, female patients demonstrated greater improvements in PNI by the sixth week compared to males, who showed only modest gains. This disparity may reflect sex-specific physiological factors, socioeconomic conditions, and the psychosocial burden of chronic kidney disease. Notably, each one-unit increase above the PNI cut-off of 37 was associated with a 2.7-fold increase in AVF maturation success in both sexes, underscoring PNI’s clinical relevance as a robust and accessible nutritional marker.

## 6. Limitations

The retrospective design of the study and the relatively small sample size may have introduced bias. Although the statistical power, calculated by G*Power analysis, reached 70%—slightly below the ideal 80% threshold—the consistency and comprehensiveness of the institutional and investigator-maintained records likely enhanced data reliability. We acknowledge that the six-week follow-up period falls short of the three-month maturation window recommended by the KDOQI guidelines [34], and that the lack of standardized criteria for successful cannulation or achieving adequate hemodialysis pump flow in pre-dialysis patients complicates the precise definition of AVF maturation. Nevertheless, close clinical monitoring with serial Doppler ultrasound assessments and evaluation of inflammatory and nutritional indices until dialysis initiation provided valuable insights into AVF hemodynamic dynamics. The high burden of comorbidities, malnutrition, and systemic inflammation inherent to CKD populations, along with potential technical variability such as laboratory equipment calibration, further challenges the attainment of definitive conclusions [79,80]. While our study presents findings on factors influencing AVF blood flow and maturation in conjunction with inflammatory and nutritional parameters, it highlights the possibility of sex-specific differences in biomarker effects and underscores the need for sex-adjusted thresholds in clinical practice. Prospective studies are clearly needed to validate these observations and to develop individualized prediction models for AVF success.

## 7. Conclusions

This study demonstrated that inflammatory and nutritional markers significantly influence AVF blood flow and maturation, with notable sex-specific differences. Female patients exhibited lower levels of albumin, iron, and vitamin D, despite being younger, while intraoperative TTFM measurements emerged as strong predictors of AVF blood flow adequacy. Elevated inflammatory indices were associated with impaired AVF maturation across both sexes. These findings emphasize the importance of considering both inflammatory status and nutritional profiles when evaluating AVF outcomes. Further prospective studies are needed to validate and expand upon these observations.

## Figures and Tables

**Table 1:** Formulas and calculation methods of indices used in the study [9,26,27].

Index/Parameter	Calculation Method
Body mass index (kg/m^2^) (BMI)	Weight (kg)/Height (m^2^)
C-reactive protein to albumin ratio (CrA)	CRP (mg/L)/Albumin (g/dL)
Neutrophil to lymphocyte ratio (NLR)	Neutrophil count/Lymphocyte count
Systemic Immune–Inflammatory Index (SII)	(Platelet count × Neutrophil count)/Lymphocyte count
Systemic Inflammatory Response Index (SIRI)	(Neutrophil count × Monocyte count)/Lymphocyte count
Prognostic Nutritional Index (PNI)	[10 × serum Albumin (g/dL)] + [0.005 × Lymphocyte count]

**Table 2:** Preoperative and intraoperative characteristics of the patients.

		Female (*n* = 50)	Male (*n* = 60)	*p*
Age (years)		50.1 ± 12.8	54.8 ± 12.9	0.061
Body mass index, (kg/m^2^)		27.7 ± 3.7	25.7 ± 2.4	0.007
Diabetes mellitus		15 (30%)	24 (40%)	0.275
Hypertension, (%)		34 (68%)	44 (73%)	0.540
Coronary artery disease, (%)		6 (12%)	22 (36.6%)	<0.01
Smoker, (%)		14 (28%)	23 (38%)	0.186
Artery diameter, mm; median (Q1–Q3)		3.8 (3.6–4.0)	3.4 (2.8–4.08)	0.013
Vein diameter, mm, median (Q1–Q3)		3.4 (3.13–3.68)	3.2 (2.9–3.6)	0.118
Transit time flow measurement	Flow (mL/min);mean	193 ± 92	282 ± 96	<0.001
	Pulsatility index	2.6 ± 1.0	1.6 ± 0.8	<0.001
Hemoglobin, g/dL		11.1 ± 1.2	11.9 ± 1.4	0.002
Neutrophil count, 10^3^/μL		5.2 ± 1.6	5.4 ± 1.5	0.490
Lymphocyte count, 10^3^/μL		1.9 ± 0.7	1.7 ± 0.6	0.114
Monocyte count, 10^3^/μL		0.6 ± 0.3	0.7 ± 0.3	0.256
Platelet count, 10^3^/μL		237 ± 65	240 ± 52	0.457
GFR, mL/min/1.73 m^2^		45.7 ± 3.8	45.3 ±3.4	0.513
Triglyceride, mg/dL		148 ± 59	145 ± 66	0.544
Albumin, g/dL		3.8 ± 0.4	3.95 ± 0.4	0.139
CRP, mg/L		12.1 ± 6.2	12.2 ± 8.7	0.228
HbA1c, %		6.0 ± 1.2	6.3 ± 1.5	0.375
Uric acid, mg/dL		5.9 ± 1.1	5.8 ± 1.2	0.401
Ferritin, ng/mL		318 ± 120	278 ± 110	0.071
Iron, µg/dL		49.3 ± 20.8	64.1 ± 26.5	0.003
Vitamin D, ng/mL		24.4 ± 14.4	34.8 ± 16.3	<0.001
CrA, median (Q1–Q3)		2.9 (1.9–3.8)	2.1 (1.6–3.5)	0.166
NLR, median (Q1–Q3)		2.5 (2.06–3.4)	3.0 (2.3–4.3)	0.040
SII, median (Q1–Q3)		598 (472–840)	704 (515–970)	0.172
SIRI, median (Q1–Q3)		1.64 (1.1–2.2)	1.86 (1.3–2.8)	0.037
PNI, median (Q1–Q3)		39 (35.5–42)	40 (37–43)	0.110

CrA, C-reactive protein to albumin ratio; CRP, C-Reactive Protein; GFR, Glomerular filtration rate; NLR, Neutrophil to lymphocyte ratio; SII, Systemic Immune–Inflammatory Index; SIRI, Systemic Inflammatory Response Index; PNI, Prognostic Nutritional Index.

**Table 3:** Dynamics of nutritional and inflammatory biomarkers over time by sex.

	Female					Male				
Parameters, Median	Preoperative	Fourth Week	Sixth Week	*p*	η^2^	Preoperative	Fourth Week	Sixth Week	*p*	η^2^
Albumin	3.9 (3.5–4.1)	3.6 (3.1–3.9)	3.8 (3.7–4.0)	<0.001	0.184	4.0 (3.7–4.2)	3.9 (3.7–4.2)	4.0 (3.8–4.2)	0.030	0.057
CRP	10.9 (7.9–14.9)	14.2 (8.8–36.8)	11.2 (6.1–40.1)	<0.001	0.279	8.9 (6.5–14.2)	12.2 (6.7–24.3)	7.1 (5.1–16.8)	0.026	0.075
Neutrophil	4.9 (3.9–6.4)	5.5 (4.3–7.2)	5.7 (4.6–7.3)	<0.001	0.261	5.5 (4.1–6.5)	5.1 (4.1–6.5)	5.6 (4.1–7.0)	0.012	0.172
Lymphocyte	1.8 (1.4–2.4)	1.3 (1.1–2.0)	1.4 (1.1–2.1)	<0.001	0.258	1.6 (1.3–2.1)	1.4 (1.0–1.8)	1.4 (1.0–1.7)	0.001	0.130
Monocyte	0.5 (0.4–0.7)	0.5 (0.4–0.8)	0.6 (0.5–0.8)	0.054	0.058	0.54 (0.4–0.7)	0.6 (0.5–0.8)	0.6 (0.4–0.8)	0.033	0.061
Ferritin	299 (236–404)	411 (263–636)	422 (300–685)	<0.001	0.307	259 (201–347)	319 (215–479)	289 (226–385)	<0.001	0.133
CrA	2.9 (1.9–3.8)	3.6 (2.2–13.0)	2.8 (1.6–10.5)	<0.001	0.287	2.1 (1.6–3.5)	3.1 (0.6–6.7)	1.7 (1.2–4.2)	0.041	0.185
NLR	2.5 (2.06–3.4)	4.6 (3.0–8.5)	4.6 (3.0–10.1)	<0.001	0.300	3.0 (2.3–4.3)	3.9 (2.6–6.5)	3.9 (2.7–6.7)	<0.001	0.175
SII	598 (472–840)	896 (591–1980)	840 (664–2354)	<0.001	0.238	704 (515–970)	964 (517–1360)	758 (568–1480)	<0.001	0.136
SIRI	1.64 (1.1–2.2)	3.0 (1.8–6.3)	3.3 (1.7–9.0)	<0.001	0.250	1.86 (1.3–2.8)	2.7 (1.5–5.2)	2.6 (1.6–4.9)	<0.001	0.173
PNI	39 (35.5–42)	36 (31–39)	38 (37–41)	<0.001	0.184	40 (37–43)	40 (37–42)	41 (39–43)	0.034	0.068

CrA, C-reactive protein to albumin ratio; CRP, C-Reactive Protein; NLR, Neutrophil to lymphocyte ratio; SII, Systemic Immune–Inflammatory Index; SIRI, Systemic Inflammatory Response Index, PNI, Prognostic Nutritional Index. *p*-values were calculated to reflect within-group comparisons based on repeated measures ANOVA or the Friedman test, as appropriate, and effect sizes were reported as partial eta squared (η^2^).

**Table 4:** Inflammatory parameters stratified by sex at the fourth and sixth weeks.

	Fourth Week			Sixth Week		
	Female	Male	*p*	Female	Male	*p*
CrA, median (Q1–Q3)	3.6 (2.2–13.0)	3.1 (.6–6.7)	0.019	2.8 (1.6–10.5)	1.7 (1.2–4.2)	0.014
NLR, median (Q1–Q3)	4.6 (3.0–8.5)	3.9 (2.6–6.5)	0.192	4.6 (3.0–10.1)	3.9 (2.7–6.7)	0.222
SII, median (Q1–Q3)	896 (591–1980)	964 (517–1360)	0.469	840 (664–2354)	758 (568–1480)	0.186
SIRI, median (Q1–Q3)	3.0 (1.8–6.3)	2.7 (1.5–5.2)	0.604	3.3 (1.7–9.0)	2.6 (1.6–4.9)	0.209
PNI, median (Q1–Q3)	36 (31–39)	40 (37–42)	0.021	38 (37–41)	41 (39–43)	0.026
Albumin, median	3.6 (3.1–3.9)	3.9 (3.7–4.2)	0.02	3.8 (3.7–4.0)	4.0 (3.8–4.2)	0.018
CRP, median	14.2 (8.8–36.8)	12.2 (6.7–24.3)	0.037	11.2 (6.1–40.1)	7.1 (5.1–16.8)	0.025

CrA, C-reactive protein to albumin ratio; CRP, C-Reactive Protein; NLR, Neutrophil to lymphocyte ratio; SII, Systemic Immune–Inflammatory Index; SIRI, Systemic Inflammatory Response Index; PNI, Prognostic Nutritional Index.

**Table 5:** Clinical predictors of AVF blood flow at the fourth and sixth postoperative weeks.

	Fourth Week		Sixth Week	
	OR (95% CI);	*p*	OR (95% CI)	*p*
Female	0.80 (0.71–0.91)	<0.001	0.81 (0.73–0.88)	<0.001
Age, years	0.93 (0.88–0.95)	0.044	0.94 (0.90–0.98)	<0.001
Body mass index, (kg/m^2^)	0.89 (0.84–0.93)	<0.001	0.91 (0.87–0.96)	0.004
Arterial diameter, mm	1.01 (0.95–1.1)	0.737	1.0(0.95–1.18)	0.680
Vein diameter, mm	1.08 (0.95–1.17)	0.789	1.02 (0.9–1.2)	0.667
TTFM mean flow	1.11 (1.03–1.4)	0.001	1.28 (1.1-.1.9)	<0.001
Diabetes mellitus	0.96 (0.86–1.06)	0.095	0.93 (0.83–1.05)	0.071
Coronary artery disease	0.93 (0.83–1.04)	0.192	0.89(0.81–0.96)	0.012
Hypertension	0.98 (0.95–1.2)	0.175	0.95 (0.89–1.01)	0.057
Current smoking	0.84 (0.76–0.95)	0.003	0.82 (0.74–0.90)	<0.001
Low Hemoglobin (<11.5 g/dL)	0.97 (0.92–1.02)	0.330	0.92 (0.8–1.03)	0.287
Albumin (≥3.85 g/dL)	1.15 (1.06–1.2)	<0.001	1.01 (0.98–1.05)	0.429
C-Reactive Protein	0.98 (0.9–1.05)	0.815	0.94 (0.84–1.01)	0.368
Vitamin D (≤ 22.7 ng/mL)	0.86 (0.79–0.97)	0.036	0.91 (0.85–0.99)	0.003

**Table 6:** Inflammatory and nutritional index-based predictors of AVF blood flow stratified by sex.

Female			Male	
Predictors	Fourth Week OR (95% CI); *p*	Sixth Week OR (95% CI), *p*	Fourth Week OR (95% CI), *p*	Sixth Week OR (95% CI), *p*
CrA	0.90 (0.78–1.28); *p* = 0.274	0.91 (0.72–1.34); *p* = 0.161	1.02 (0.98–1.08); *p* = 0.097	1.08 (0.86–1.3); *p* = 0.150
NLR	0.68 (0.40–0.98); *p* = 0.044	0.68 (0.34–0.95); *p* = 0.020	1.07 (0.98–1.12); *p* = 0.125	1.01 (0.82–1.1); *p* = 0.379
SII	1.1 (0.91–2.2); *p* = 0.202	1.1 (0.8–2.0); *p* = 0.598	0.73 (0.4–1.13); *p* = 0.077	0.85 (0.5–1.3); *p* = 0.304
SIRI	1.2 (0.8–1.7); *p* = 0.152	1.3 (0.95–2.1); *p*= 0.105	0.79 (0.59–0.97); *p* = 0.049	0.73 (0.5–0.98); *p* = 0.005
PNI	0.91 (0.8–1.03); *p* = 0.115	1.12 (0.92–1.4); *p* = 0.084	0.98 (0.9–1.12); *p* = 0.466	1.2 (0.9–1.8); *p* = 0.332

CrA, C-reactive protein to albumin ratio; NLR, Neutrophil to lymphocyte ratio; SII, Systemic Immune–Inflammatory Index; SIRI, Systemic Inflammatory Response Index; PNI, Prognostic Nutritional Index.

**Table 7:** Negative predictors of AVF maturation.

Predictor	Multivariate Regression OR (95% CI)	*p*-Value
Female sex	1.7 (1.15–12)	0.037
Advanced age	1.3 (1.1–1.61)	0.023
Obesity	1.4 (1.3–1.86)	<0.001
Diabetes	1.2 (1.1–1.37)	0.001
Hypertension	1.2 (1.17–1.39)	0.004
Current smoking	1.9 (1.3–1.61)	0.02
Hemoglobin ≤ 11.5 g/dL	1.7 (1.3–2.5)	0.012
Vitamin D ≤ 22.7 ng/mL	1.3 (1.05–1.91)	<0.01
NLR	1.38 (1.1–1.32)	0.004
SII	1.5 (1.1–2.4)	0.018
SIRI	2.3 (1.2–5.0)	0.021
CrA	1.6 (0.8–3.3)	0.257

CrA, C-reactive protein to albumin ratio; NLR, Neutrophil to lymphocyte ratio; SII, Systemic Immune–Inflammatory Index; SIRI, Systemic Inflammatory Response Index.

## Data Availability

All data generated or analyzed during this study, including imaging materials, laboratory findings, and statistical data, are securely stored within the authors’ archives. No data were obtained from external sources or previously published materials. These datasets are available from the corresponding author upon reasonable request.

## Abbreviations

AVF	Arteriovenous fistula
BMI	Body mass index
CKD	Chronic kidney disease
CrA	C-reactive protein to albumin ratio
CRP	C-reactive protein
DUS	Doppler ultrasonography
GFR	Glomerular filtration rate
HD	Hemodialysis
NLR	Neutrophil-to-lymphocyte ratio
PNI	Prognostic nutritional index
SII	Systemic Immune–Inflammatory Index
SIRI	Systemic inflammation response index
TTFM	Transit-Time Flow Measurement
